# Supplementary material for: Does local government tax competition promote industrial transformation and upgrading?
Source: PLoS One. 2025 Dec 19;20(12):e0335886. doi: 10.1371/journal.pone.0335886 (PMC12716791; doi:10.1371/journal.pone.0335886)
Supplement: Attachment — Submitted filename: Response to Reviewers.docx [file pone.0335886.s003.docx]

**Responses to Reviewers**

Dear Editors and Reviewers,

Thank you very much for carefully reading the manuscript. We are very grateful to your helpful and valuable recommendations for further improvements of the paper. We have revised the manuscript according to all of the reviewers’ and editors' comments and suggestions. All the revised contents have been marked red in the manuscript, and the answers to the reviewers’ comments have been marked dark blue in this response letter. More details are as follows.

**Reviewers1:**

Comments

This article systematically explains the nonlinear impact and transmission mechanism of industrial transformation and upgrading from the perspective of local tax competition, and tests the regulatory effect of technological innovation between the two, which has very important research value and significance for promoting high-quality economic development. However, it is undeniable that the thesis still has room for improvement, and there are some flaws and deficiencies that need to be further supplemented and improved:

(1) The second paragraph of Section 3.2 describes the negative impact on industrial transformation and upgrading when the intensity of local government tax competition exceeds a reasonable threshold. Local governments should further reduce the proportion of direct taxes when the intensity of tax competition exceeds a reasonable threshold. However, the sentence "however, the move by local governments to increase the proportion of direct taxes has directly squeezed corporate profit margins, weakening their ability to invest in research and development, equipment upgrades, and technical innovation." in the fifth line of this paragraph indicates that local governments should increase the proportion of direct taxes, which is contrary to the previous view.

R: We are most grateful for the reviewer's valuable comments. We fully acknowledge the importance of theoretical consistency for the rigour of the paper. To this end, we have refined the section on the mechanisms of direct taxation and tax competition, significantly enhancing the theoretical coherence of the relevant discussion. (p.6-p.8)

(2) Grammar needs further polishing.

R: We are most grateful for the reviewer's valuable comments. We fully acknowledge that the manuscript requires refinement in its linguistic expression. To this end, we have engaged a native English-speaking professional peer to conduct a systematic linguistic polish of the entire text. We have systematically reviewed each paragraph to identify and eliminate redundant content, thereby ensuring compliance with the linguistic standards of academic journals.

(3) It is also suggested that the calculation of control variables should also include references to relevant literature.

R: We are most grateful for the reviewer's valuable comments. We fully appreciate that in academic research, providing references for the description and computational methods of control variables enhances the rigour and reproducibility of the study, while also demonstrating the scholarly rationale for methodological choices. Accordingly, we have supplemented each control variable mentioned in the text with the relevant references. (p.13-p.14)

(4) In the "Benchmark regression results", for the linear part, if the level of local government tax competition increases by 1 unit, how many more units will industrial transformation and upgrading increase?.

R: We are most grateful for the reviewer's valuable comments. We fully concur that the clarity of regression result interpretations is crucial for enhancing the readability of research. To this end, we have supplemented the regression coefficients for the core explanatory variables with specific quantitative interpretations, particularly regarding the magnitude and meaning of the coefficients. (p.15)

(5) It is recommended to use English references instead of Chinese references.

R: We are most grateful for the reviewer's valuable suggestions. We fully concur that prioritizing English-language references in international academic publications constitutes a crucial principle for enhancing research traceability and ensuring accessibility for an international readership. This recommendation is accorded the utmost importance. The primary reason for retaining this non-English reference in our study lies in the unique comprehensiveness of its proposed methodology for measuring industrial transformation and upgrading. This approach serves as an indispensable cornerstone supporting our research's computational logic, rendering it irreplaceable. To adequately balance the necessity of the citation with the readability requirements of an international journal, we have provided a detailed explanation of the specific measurement methodology for industrial transformation and upgrading from the non-English source within the original text. This ensures international readers can accurately grasp its core concepts and role within this study. (p.12)

(6) It is suggested that the explanations for "lnno", "Low lnno" and "High lnno" in Figure 3 be placed in the footnotes.

R: We are most grateful for the reviewer's valuable comments. We fully appreciate the importance of clear explanations for chart elements in enhancing data readability, and acknowledge that certain figures in the original manuscript (such as Figure 3) lacked sufficient detail in their explanations regarding variable definitions, which may have hindered readers' accurate understanding of the results. Accordingly, we have added detailed explanations for all variables featured in Figure 3 within the caption text, ensuring their conceptual clarity. (p.23)

**Reviewers2:**

(1) The text in the article could be further refined.

R: We are most grateful for the reviewer's valuable comments. We fully acknowledge that the manuscript requires refinement in its Verbal expression. To this end, we have engaged a native English-speaking professional peer to conduct a systematic linguistic polish of the entire text. We have systematically reviewed each paragraph to identify and eliminate redundant content, thereby ensuring compliance with the linguistic standards of academic journals.

(2) The language could be further refined.

R: We are most grateful for the reviewer's valuable suggestions. We have streamlined and refocused the language throughout the text to more clearly underpin the research value of this study. (p.4-p.6)

(3) It is recommended that all references be in English.

R: We are most grateful for the reviewer's valuable suggestions. The primary reason for retaining this non-English reference in our study lies in the unique comprehensiveness of its proposed methodology for measuring industrial transformation and upgrading. This approach serves as an indispensable cornerstone supporting our research's computational logic, rendering it irreplaceable. To adequately balance the necessity of the citation with the readability requirements of an international journal, we have provided a detailed explanation of the specific measurement methodology for industrial transformation and upgrading from the non-English source within the original text. (p.12)

(4) References can also be included in the description of controlled variables.

R: We are most grateful for the reviewer's valuable comments. We fully appreciate that in academic research, providing references for the description and computational methods of control variables enhances the rigour and reproducibility of the study, while also demonstrating the scholarly rationale for methodological choices. Accordingly, we have supplemented each control variable mentioned in the text with the relevant references. (p.13-p.14)

(5) The literature review should be more concise and focused.

R: We are most grateful for the reviewer's valuable suggestions. We fully appreciate the importance of conciseness and focus in the literature review for enhancing the logical coherence of the paper. Accordingly, we have streamlined and refocused this section to more clearly underpin the research value of this study. (p.4-p.6)

6) There are several repetitive expressions in the writing of the article. It is suggested that the author further simplify the language.

R: We are most grateful for the reviewer's valuable comments. We have reviewed the entire text, removed redundant expressions, and further simplified the language.
